# Supplementary material for: The individualized neural tuning model: Precise and generalizable cartography of functional architecture in individual brains
Source: Imaging Neurosci (Camb). 2023 Nov 22;1:imag-1-00032. doi: 10.1162/imag_a_00032 (PMC11501089; doi:10.1162/imag_a_00032)
Supplement: Supplementary Material [file imag_a_00032-supp.pdf]

Supplementary Materials

1. Comparison between hyperalignment algorithms

The INT model is based on the new hyperalignment algorithm—warp hyperalignment. Different from the classic hyperalignment (Guntupalli et al., 2016; Haxby et al., 2020, 2011), warp hyperalignment uses ridge regression in place of the orthogonal Procrustes transformation, and it incorporates ensemble learning into the model. Therefore, it is of interest to compare the performance of the INT model with different hyperalignment algorithms.

In the INT model, the neural tuning of each individual is modeled as an individualized transformation applied to a common functional template. We systematically evaluated its performance in five different ways to derive the transformation:

- (a) warp hyperalignment with ensemble averaging (same as the manuscript),
- (b) warp hyperalignment without ensemble averaging (i.e., single model),
- (c) Procrustes hyperalignment with ensemble averaging,
- (d) Procrustes hyperalignment without ensemble averaging,
- (e) no hyperalignment (i.e., identity transformation from template to subject).

These comparisons were performed on the same data, and different conditions only differed in the hyperalignment algorithm used.

Specifically, we assessed the performance of each model using six different benchmarks, repeated for each individual (i.e., leave-one-participant-out cross-validation):

1. The correlation between the measured and model-predicted time series of each cortical vertex;

2. The correlation between measured and predicted representational dissimilarity matrix (RDM) in each searchlight (10 mm radius);
3. The correlation between measured and predicted object category selectivity maps;
4. The correlation between measured and predicted retinotopic maps;
5. The correlation of measured and predicted brain response pattern for each time point of the movie;
6. The multiclass movie time point classification accuracy based on the entire cortex.

The better-performing algorithm is expected to yield higher similarities between measured and predicted time series, RDMs, category-selectivity and retinotopic maps, and response pattern to the movies, as well as higher classification accuracy.

We found very consistent results across different benchmarking indices. Specifically, warp hyperalignment models, both with and without ensemble averaging, perform the best among all models. Warp hyperalignment with ensemble averaging worked slightly better than without ensemble averaging, yet the difference was often small, which suggests that ensemble averaging can be skipped for warp hyperalignment when computational resources are limited. All four hyperalignment models perform much better than the control model without hyperalignment, reiterating the importance of resolving idiosyncrasies in functional–anatomical correspondence with hyperalignment algorithms. The performance for Procrustes hyperalignment algorithms was not as good as warp hyperalignment, but still much better than the control model. Interestingly, Procrustes hyperalignment with ensemble averaging worked better than Procrustes hyperalignment without ensemble averaging. Mathematically, the average of multiple orthogonal matrices is not necessarily an orthogonal matrix. Therefore, the transformation matrices based on ensemble Procrustes hyperalignment, each of which is an average transformation, might be less constrained by orthogonality and more similar to the

transformation matrices based on warp hyperalignment. In short, the model performance can be summarized as:

WHA ensemble > WHA single >> ProcrHA ensemble >> ProcrHA single >> No HA

where WHA is short for warp hyperalignment, ProcrHA is short for Procrustes hyperalignment, and ensemble and single refer to models with and without ensemble averaging, respectively.

In the figures below, we use green colors for warp hyperalignment, blue colors for Procrustes hyperalignment, and orange for the control condition (no hyperalignment). Within each hyperalignment algorithm, the ensemble model is denoted using darker color, and the single model is denoted using lighter color.

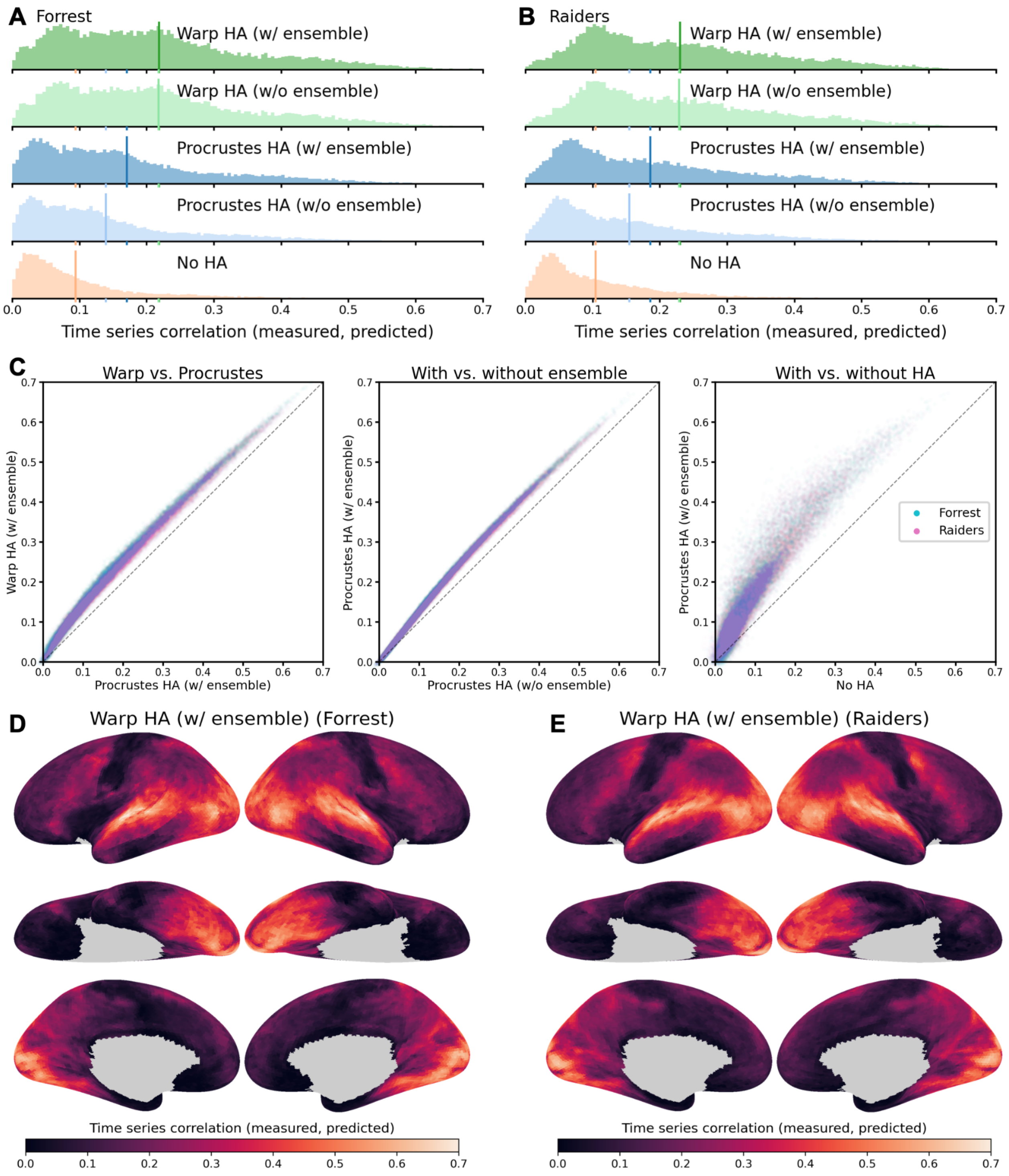

Figure S1. Comparison of model performance based on time series correlation. In this analysis, we correlated the measured and model-predicted response time series to the movie for each vertex, each subject, and each model. If a model performs better than others, we expect its predicted time series will have higher correlation with the measured time series. (A

and B) The distribution of correlation coefficients based on five different methods for the Forrest dataset and the Raiders dataset, respectively. (C) Scatter plots comparing warp hyperalignment and Procrustes hyperalignment (left), Procrustes hyperalignment with and without ensemble averaging (middle), and Procrustes hyperalignment and no hyperalignment (right), which allows accessing the effects of warping representational geometry, using ensemble averaging, and using hyperalignment, respectively. (D and E) The distribution of correlation coefficients on the cortical surface based on warp hyperalignment with ensemble averaging for the Forrest dataset and the Raiders dataset, respectively. The correlation was high for both early and association visual and auditory cortices, as well as high-level cognitive areas.

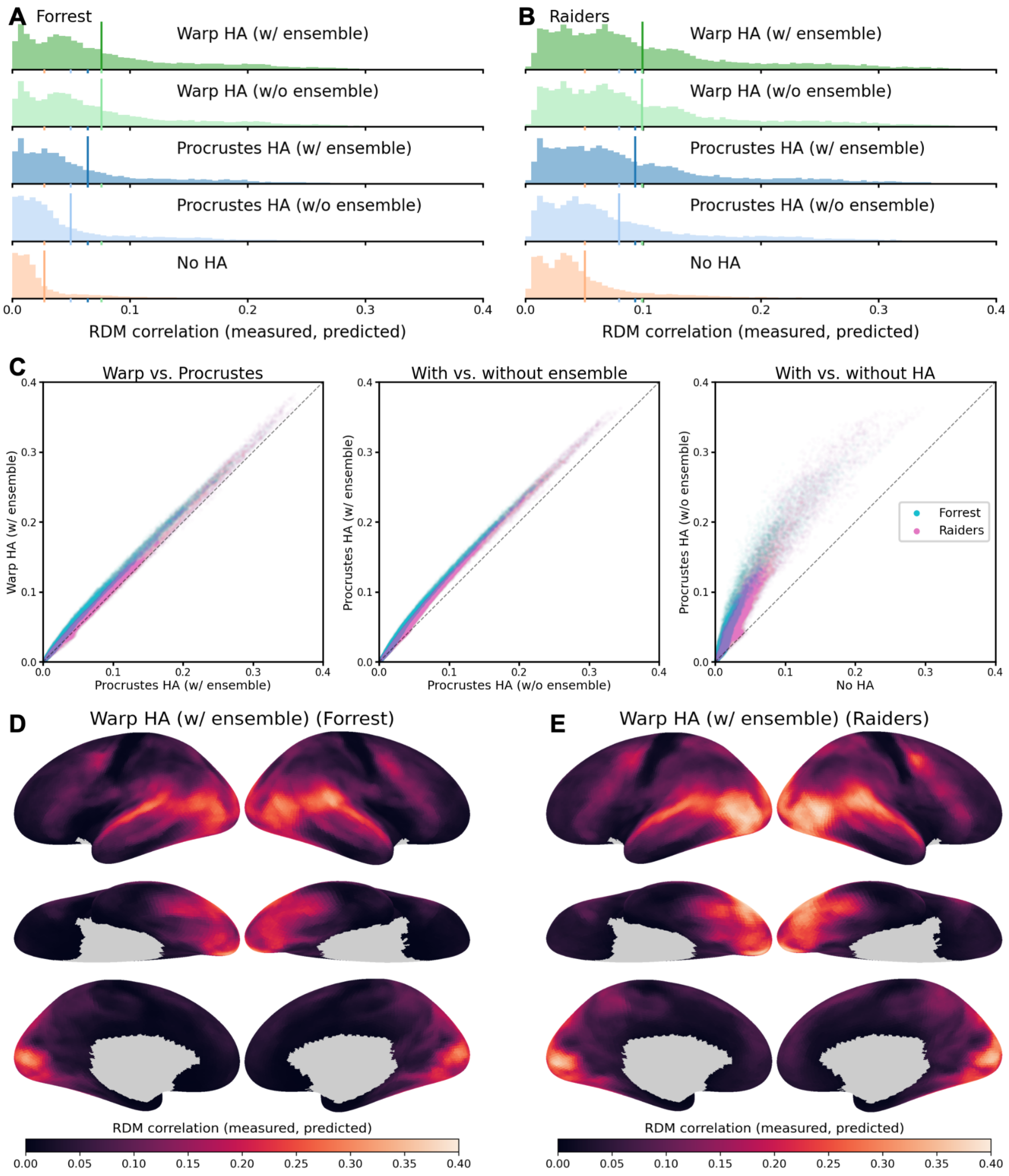

Figure S2. Comparison of model performance based on RDM correlation. In this analysis, for each 10 mm searchlight, we correlated the RDMs computed using measured and model-predicted response patterns, which was repeated for each subject and each model. If a model performs better than others, we expect the RDM based on its prediction to be more

similar to the measured RDM. The analysis differs from the one above in that it compares RDMs based on a searchlight instead of time series based on a vertex. (A and B) The distribution of searchlight RDM correlations based on five different methods for the Forrest dataset and the Raiders dataset, respectively. (C) Scatter plots comparing warp hyperalignment and Procrustes hyperalignment (left), Procrustes hyperalignment with and without ensemble averaging (middle), and Procrustes hyperalignment and no hyperalignment (right), which allows accessing the effects of warping representational geometry, using ensemble averaging, and using hyperalignment, respectively. (D and E) The distribution of RDM correlations on the cortical surface based on warp hyperalignment with ensemble averaging for the Forrest dataset and the Raiders dataset, respectively.

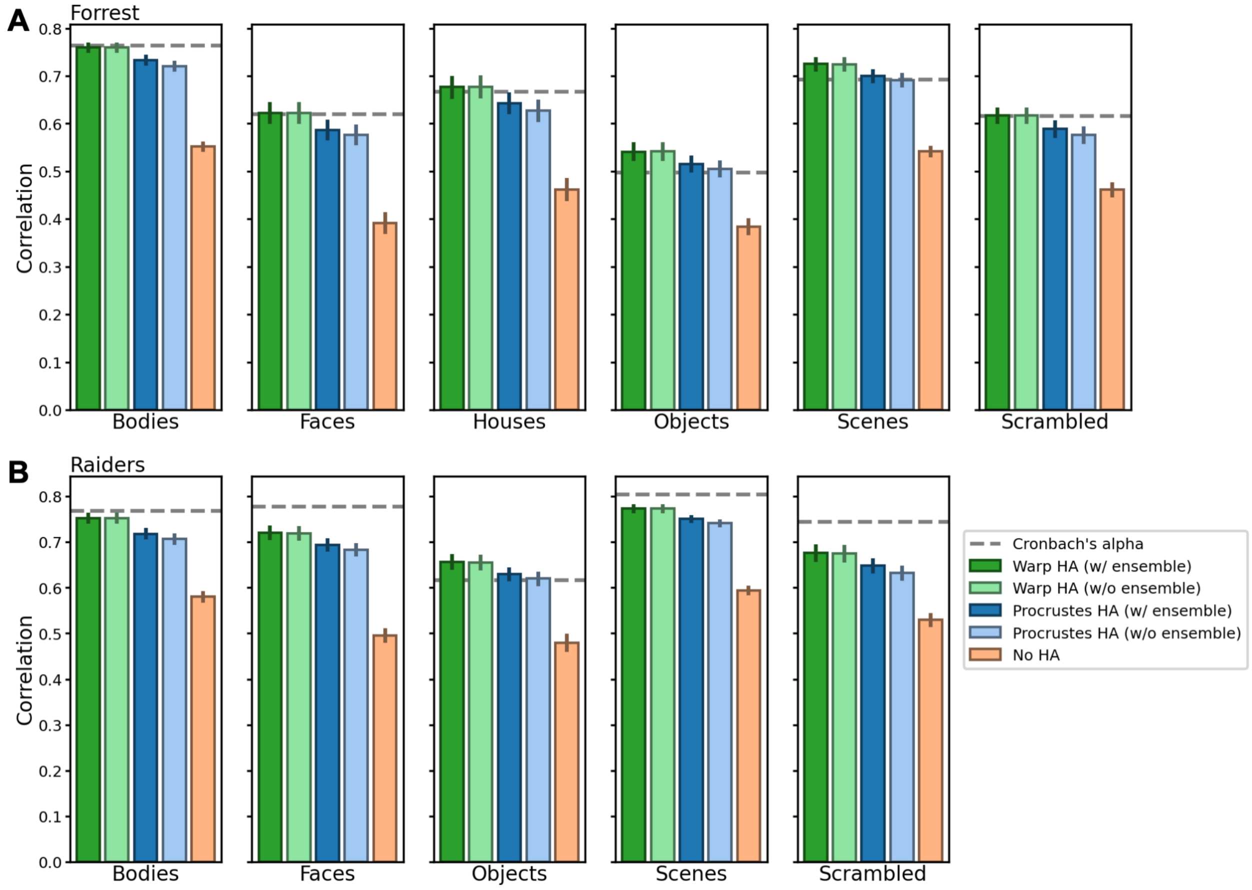

Figure S3. Comparison of model performance based on predicting category-selectivity maps. In this analysis, for each model and each subject, we trained the model using the movie data and used the obtained tuning matrices and other subjects' category-selectivity maps to predict the test subject's category-selectivity maps. If a model performs better than others, we expect the correlation between the measured maps (based on localizer scans) and the mode-predicted maps to be high. (A) Average correlation of category-selectivity maps for the Forrest dataset. The six panels are the correlations for the selectivity maps of bodies, faces, houses, objects, scenes, and scrambled objects, respectively. (B) Average correlation of category-selectivity maps for the Raiders dataset. The five panels are the correlations for the selectivity maps of bodies, faces, objects, scenes, and scrambled objects, respectively. Note that the localizers for the Forrest dataset were based on static images, and those for the Raiders dataset were based on dynamic video clips. Error bars denote standard error of the mean.

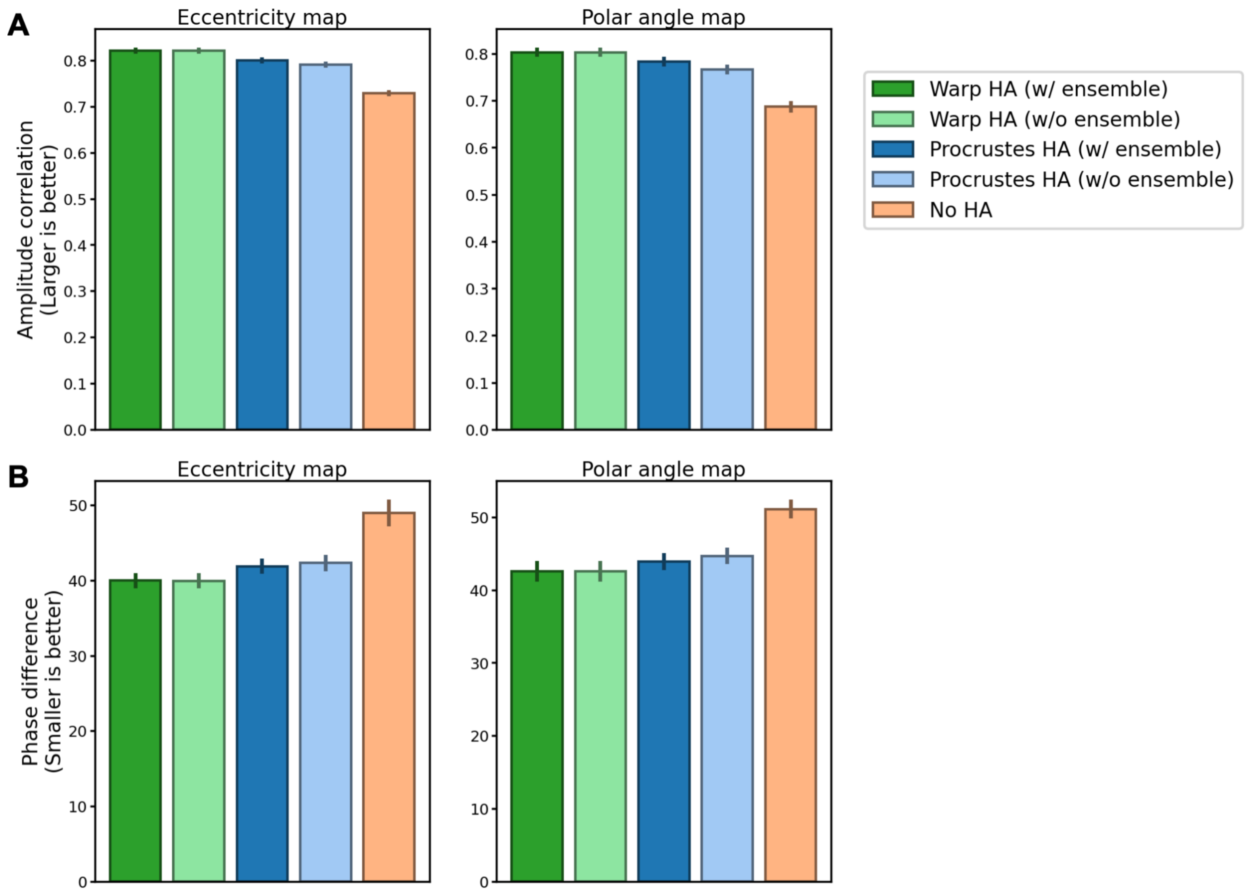

Figure S4. Comparison of model performance based on predicting retinotopic maps. In this analysis, for each model and each subject, we trained the model using the movie data and used the obtained tuning matrices and other subjects' retinotopic maps to predict the test subject's retinotopic maps. If a model performs better than others, we expect the correlation between the measured amplitude maps (based on retinotopic scans) and the mode-predicted amplitude maps to be high (Panel A), and the difference between measured and predicted phase maps to be low (Panel B). Error bars denote standard error of the mean.

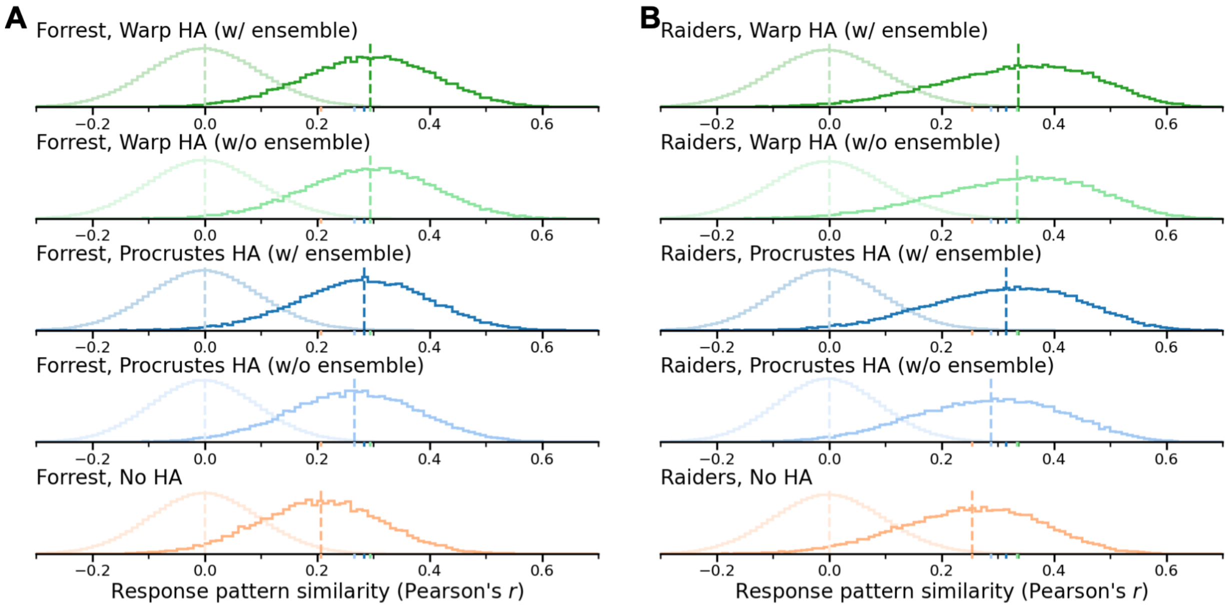

Figure S5. Comparison of model performance based on pattern similarity. In this analysis, we trained the INT model based on the first half of the movie and used it to predict the response patterns to the second half of the movie. For each subject and each time point, we computed the correlation between the measured and predicted response patterns. If a model performs better than others, we expect the pattern correlations based on the model to be higher. The dotted vertical line was the average correlation for each model. Similar to the main manuscript, we also computed the correlation between response patterns to different time points as a control analysis (lines in faded color), and these correlations always centered around 0.

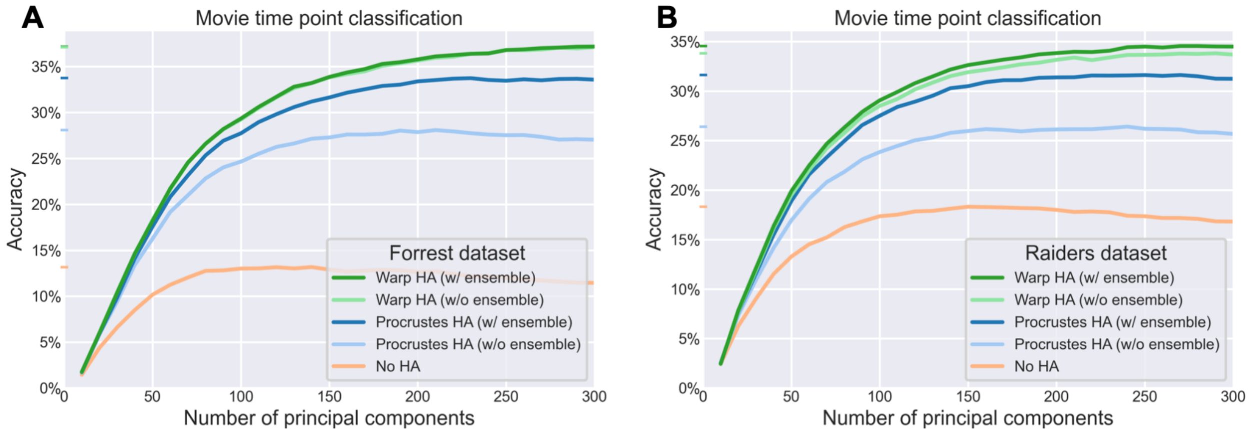

Figure S6. Comparison of model performance based on movie time point classification. In this analysis, we used the similarity between measured and predicted response patterns to predict which time point of the movie the subject was watching. Specifically, we compared the measured response pattern to a given time point of the movie to all predicted patterns of the subject (> 1000) to see whether the correlation between the measured and predicted patterns of the same time point was the highest among all predicted patterns. If a model performs better than others, we expect the classification accuracy based on the model to be higher. To facilitate the comparisons, the analysis slightly differs from the one in the main text. Specifically, the k-fold bagging method enables estimating model performance based on training data using out-of-bag cross-validation. In other words, we were able to estimate which vertices can be better predicted by the ensemble model. Weighting the vertices based on out-of-bag performance could increase classification accuracy. However, this option is only available for the ensemble models. Therefore, for a fair comparison between ensemble models, single models, and the control model, we removed vertex-weighting of the ensemble models, so that the differences in performance were only caused by differences between models.

2. Template quality as a function of the number of training participants

In the figure below, we show that the quality of the functional template continuously improves with more training participants used to build the template.

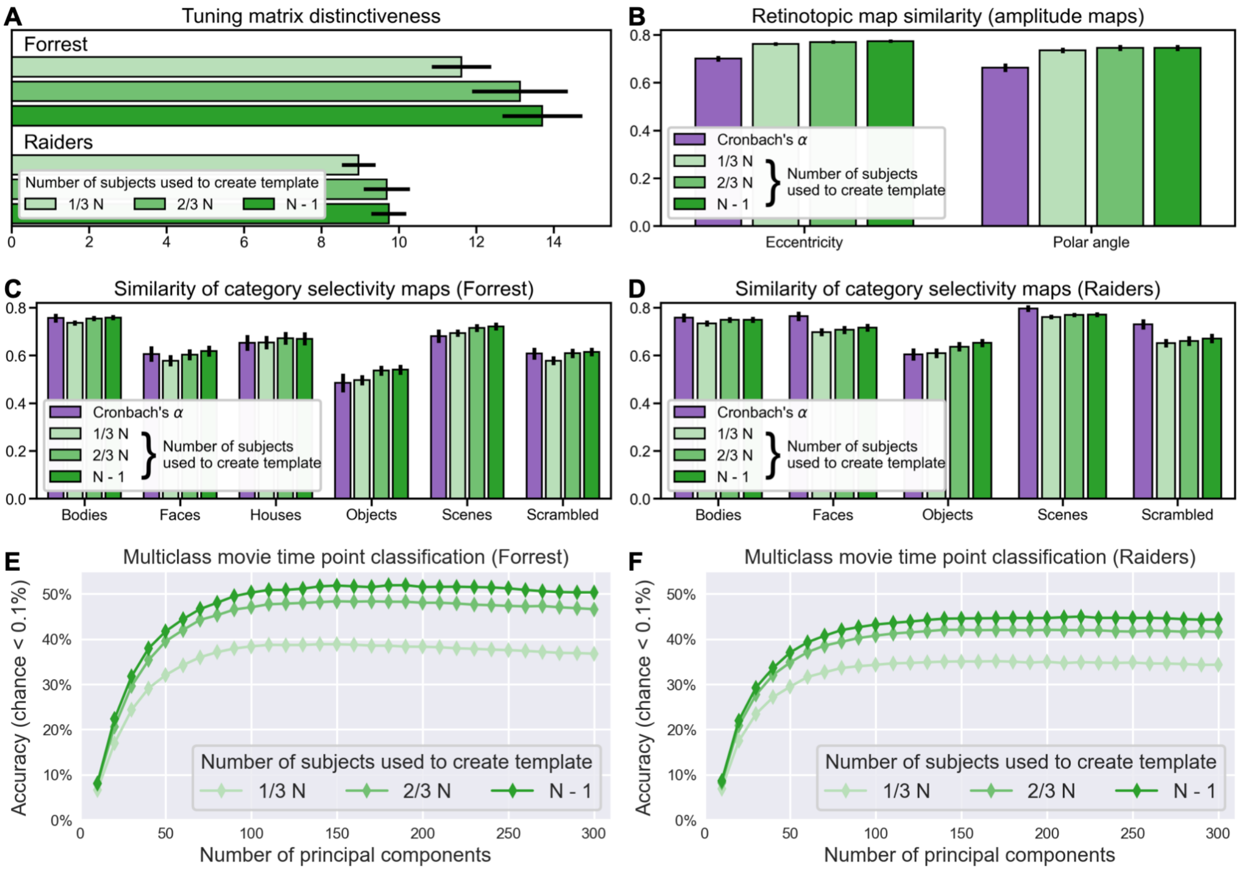

Figure S7. Template quality as a function of the number of training participants. The first step of our INT model re-represents each individual's neural response data matrix as a functional template linearly transformed with an idiosyncratic transformation, and thus the quality of the functional template may affect the performance of our INT model. We systematically manipulated the number of the participants used to create the functional template (i.e., training participants) and evaluated the performance of our INT model as a function of the number of training participants. We found that the performance of our INT model consistently increased with more training participants used to create the functional template. We observed the performance increase for the distinctiveness index **(A)**, the prediction of retinotopic maps **(B)** and category selectivity maps **(C–D)**, and the prediction of response patterns to movie time points **(E–F)**. This suggests that future development of the INT model may benefit from building the functional template based on more training participants than the current study (*Forrest*: N = 15; *Raiders*: N = 23). Error bars denote the standard error of the mean.

3. Measured and model-predicted face selectivity and retinotopic maps for all participants

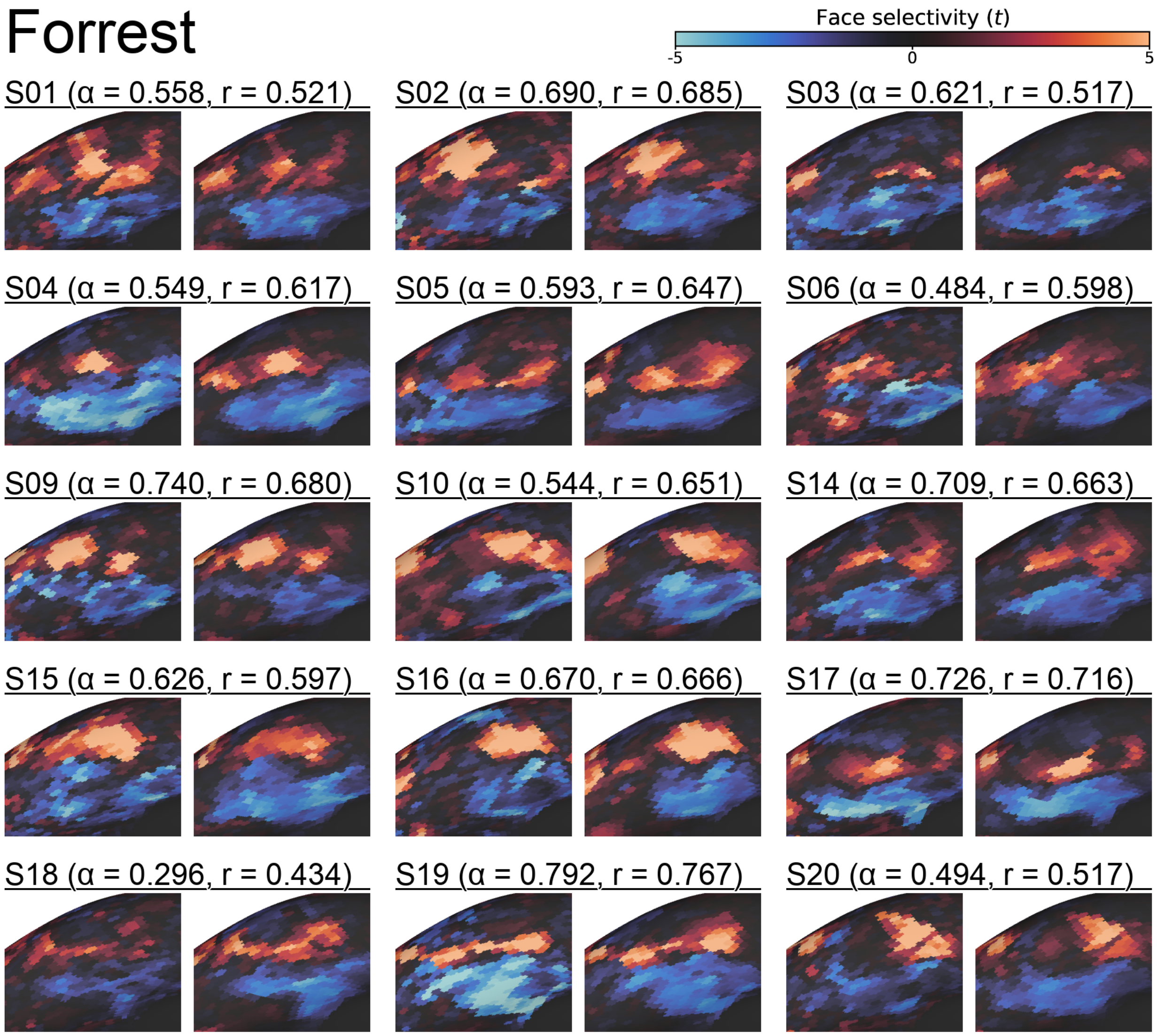

Figure S8. Face selectivity maps for all *Forrest* participants. For each participant, the estimated face selectivity map based on localizer scans is shown on the left, and the estimated map based on the INT model is shown on the right. Similar to Figure 3, the zoomed-in view of the right ventral temporal cortex is shown. α is Cronbach's alpha coefficient for the localizer-based maps, and r is the Pearson correlation between localizer-based and model-predicted maps. Both α and r are based on whole-brain face selectivity maps.

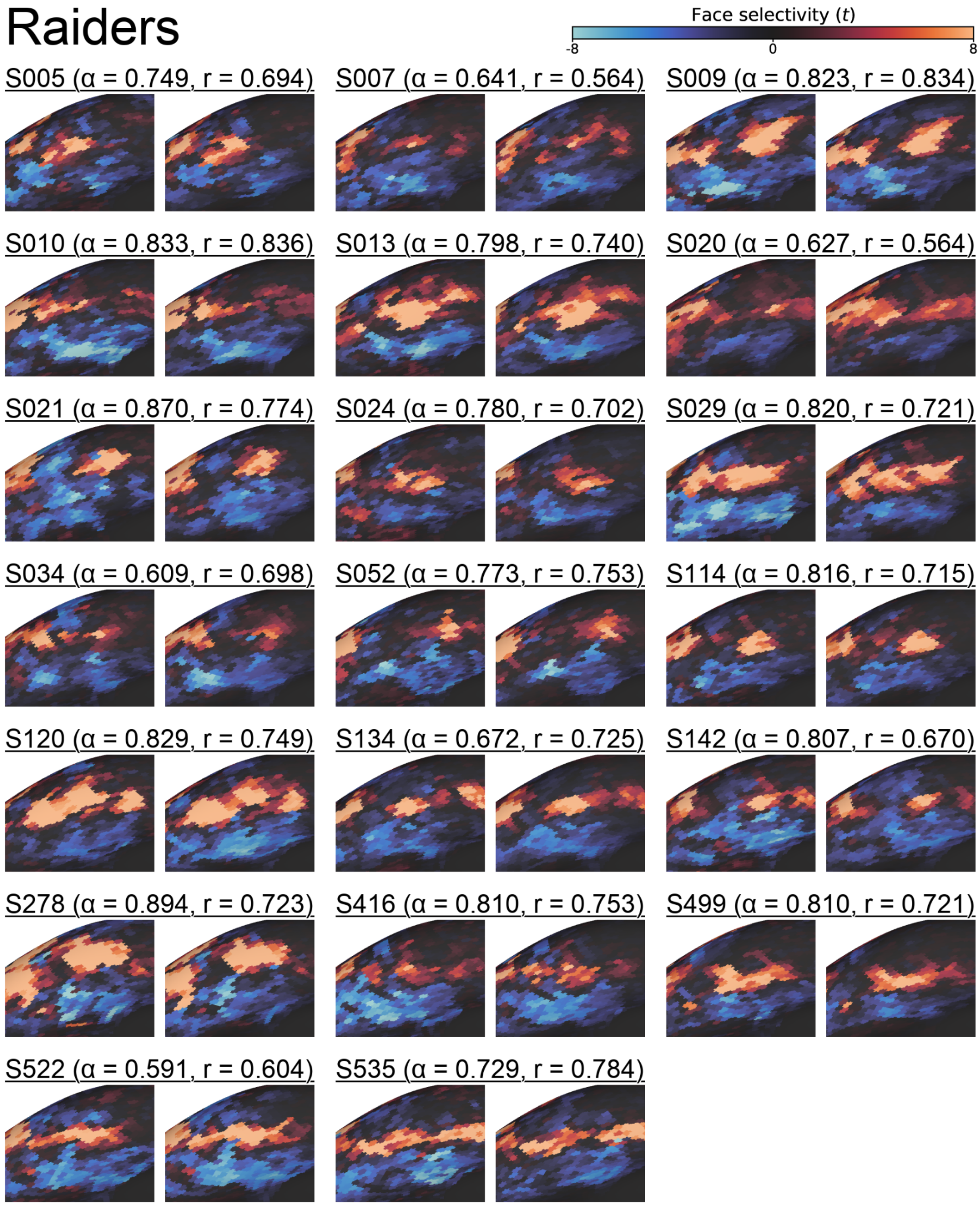

Figure S9. Face selectivity maps for all *Raiders* participants. For each participant, the estimated face selectivity map based on localizer scans is shown on the left, and the estimated map based on the INT model is shown on the right. Similar to Figure 3, the zoomed-in view of the right ventral temporal cortex is shown. α is Cronbach's alpha coefficient for the

localizer-based maps, and r is the Pearson correlation between localizer-based and model-predicted maps. Both α and r are based on whole-brain face selectivity maps.

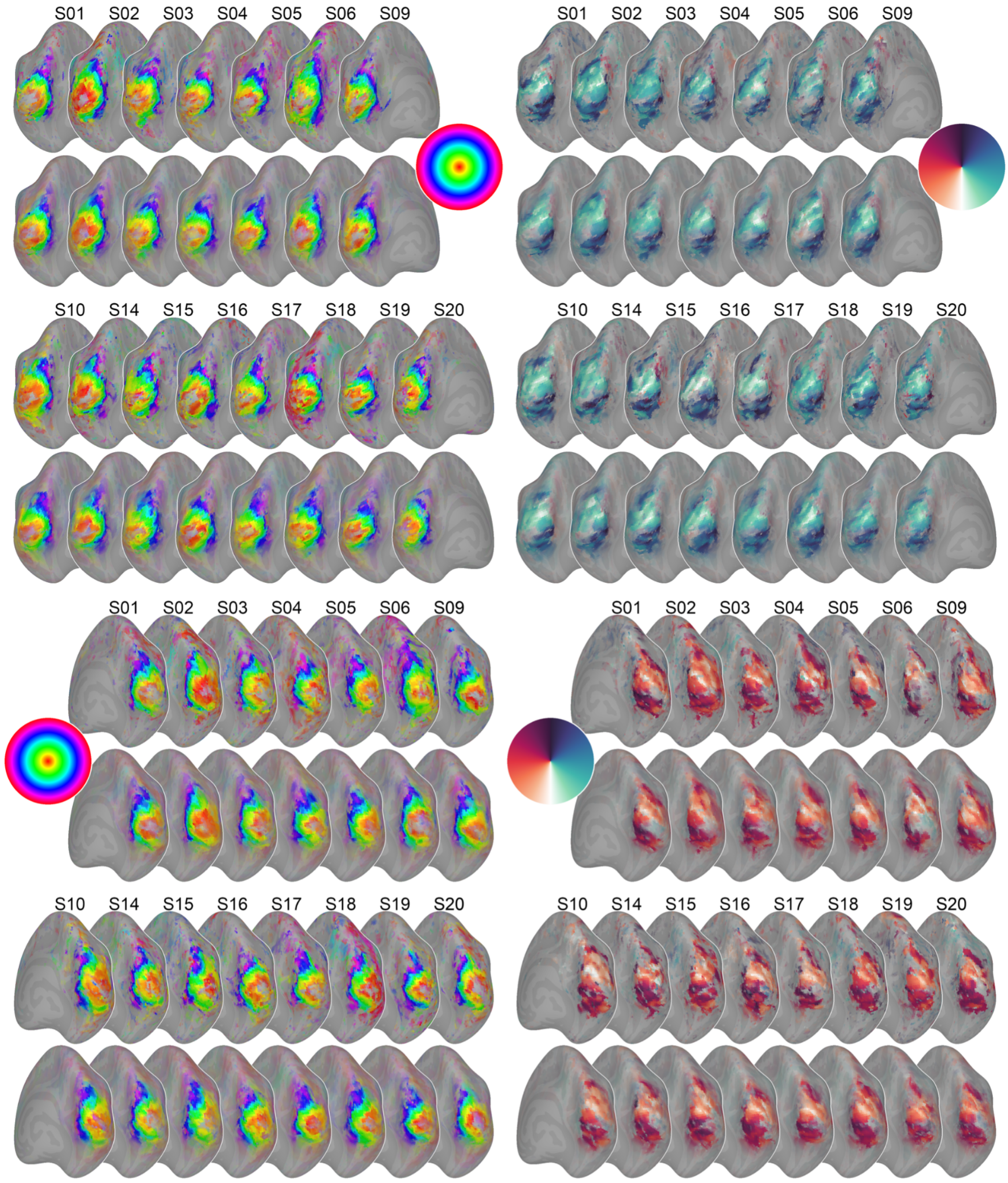

Figure S10. Estimated retinotopic maps for all participants. For each participant, the localizer-based map is shown in the top row, and the model-predicted map is shown in the bottom row. The retinotopic eccentricity maps are on the left side, and the polar angle maps are on the right side.
